# Learning Curve of Real-Time Imaging with C-Arm Based Tomography for Peripheral Lung Nodule Biopsy

**DOI:** 10.3390/life13040936

**Published:** 2023-04-03

**Authors:** Grant Senyei, Matthew Nobari, Russell Miller, Brody Harrell, George Z. Cheng

**Affiliations:** 1 Division of Pulmonary, Critical Care, and Sleep Medicine, University of California San Diego, La Jolla, CA 92037, USAgcheng@health.ucsd.edu (G.Z.C.); 2 Department of Pulmonary Medicine, Naval Medical Center San Diego, San Diego, CA 92134, USA; 3 California State University, San Bernadino, CA 92407, USA

**Keywords:** learning curve, navigational bronchoscopy, interventional pulmonology

## Abstract

The number of procedures required to attain proficiency with new bronchoscopic biopsy technologies for peripheral pulmonary lesions (PPLs) is uncertain. A prospective, single-center study evaluated learning curves of two operators performing PPL biopsies using a novel, real-time, intraoperative tomographic imaging system in consecutive procedures in adults with CT-detected PPLs. Operators were considered “proficient” when they asked three or fewer questions of the manufacturer’s clinical representative with no subsequent navigations in which they asked more than three questions. A total of 31 procedures were performed on 31 patients (Operator 1: 18, Operator 2: 13). Proficiency was achieved after an average of 10 procedures (Operator 1: 12, Operator 2: 8). From the learning curve to the post-learning curve period, the number of questions (median [IQR]: 23 [9.5–41.5] versus 0 [0–1], *p* < 0.001) and radiation dose (median [IQR]: 19.5 mGy/m^2^ [1.9–43.5] versus 1.5 mGy/m^2^ [0.7–3.3], *p* = 0.05) decreased significantly; procedure time decreased (median [IQR]: 12 min [7–20] versus 8 min [3–15], *p* = 0.29); and diagnostic yield increased significantly (13/20 cases [65%] to 11/11 cases [100%]), (*p* = 0.03). Based on this unique, clinically relevant method of assessing learning curve, proficiency with the Body Vision system was achieved at approximately the tenth procedure. These findings require validation in larger, diverse populations.

## 1. Introduction

Early, accurate diagnosis of peripheral pulmonary lesions (PPLs) is essential for detecting early stage lung cancer when it is most treatable. Early treatment of non-small-cell lung cancer dramatically increases 5-year survival rates for small, localized lesions [1,2]. With the use of low-dose computed tomography (CT), small, indeterminate lung lesions and pulmonary nodules are increasingly discovered through lung cancer screening programs [3,4] and incidentally noted on CT scans performed for other reasons [5]. Navigating to these suspicious lesions can be difficult. Lesion size and location can limit the ability of operators to acquire samples in bronchial generations beyond direct visualization of conventional flexible bronchoscopy. Additionally, obtaining an accurate biopsy in the complex and dynamic environment of the lung has been limited by low diagnostic yield with conventional bronchoscopy [6]. CT-guided transthoracic needle/core biopsy achieves higher yield, but at a cost of higher complication rates, including pneumothorax and inability to perform mediastinal staging [7,8,9,10].

Over the last two decades, a number of new navigational bronchoscopy platforms and real-time, intraoperative visualization technologies have made it possible to reach and biopsy smaller PPLs [9,11,12,13]. Used in various combinations, techniques, such as virtual bronchoscopy, electromagnetic navigation bronchoscopy (ENB), and radial endobronchial ultrasound (rEBUS), have increased diagnostic yield to the level of 70%. The advent of intraoperative computational tomographic visualization platforms, such as cone-beam computed tomography (CBCT) and C-arm-based computed tomography (CABT) systems, combined with augmented fluoroscopy have increased diagnostic yield to about 90% [6].

A learning curve refers to the process of acquiring a new technical skill and the rate at which the performance of that skill or technique improves over time. The concept of the learning curve has been applied to various medical scenarios, from surgery to diagnostic procedures.

The learning curve can be defined as the relationship between the number of procedures performed and the corresponding performance measures and outcomes, such as time to completion, rate of complications, or diagnostic yield. Typically, at the beginning of the learning curve, a novice operator’s performance is substandard, leading to higher rates of complications or longer procedural times. As the operator becomes more experienced, the performance steadily improves, and the learning curve reaches a plateau as the operator approaches proficiency.

Complexity of the procedure, the prior experience of the operator, and the learning environment can all impact the shape of the learning curve. For procedures that have a steep learning curve, there is a significant improvement in skill after only a small number of cases. Meanwhile, procedures that have a flatter learning curve require a large number of cases before operators see any significant improvement in skill. 

A key utility of learning curves is the ability to identify the number of procedures required for an operator to achieve proficiency. Proficiency can be defined as a specific outcome or complication rate that is acceptable and associated with more experienced operators. As such, the shape of a learning curve can help to identify where this proficiency is attained by reaching the flatter portion of the curve. Additionally, learning curves and identification of proficiency inflection points can help design training programs and guide the design and implementation of quality control monitoring. 

Accurate creation of a learning curve requires a standardized method of assessing performance. This standardization can involve tracking the number of procedures performed, required procedural time, any adverse events or complications, or results and outcomes of the procedure. Standardized tools and checklists can be helpful by creating unbiased measurement and recording capabilities to assess operators’ procedural skills and determine the frequency of complications. 

Understanding the learning curve for a given procedure or technology can have significant implications related to patient safety and outcomes. As an operator begins to learn a new technology, they are often on the steep part of the learning curve, and, as such, may be at greater risk of adverse events or complications. At this point, it may benefit patients to have a more experienced provider either perform or proctor the other operator during this phase. Additionally, once an operator has achieved proficiency and their learning curve has plateaued, there is an opportunity to implement quality control metrics to ensure the operator’s performance remains at a high level and any deviations can be identified and rectified quickly. 

The learning curve is a critical component related to training and adoption of new technology [14]. By examining the learning curve of a given procedure, educators and operators can develop effective programs for training, design and implement quality control measures, and improve the safety and reliability of care provided to patients.

When incorporating a novel technology, the learning curve will influence both workflow efficiency and clinical outcomes. However, limited data currently exist regarding the learning curve required to become proficient in these procedures. Although it is important to have an accurate estimate of the number of procedures required to achieve proficiency for training and credentialing purposes, there is no consensus on which measures (e.g., procedure time, diagnostic yield, etc.) should be used to establish a learning curve or determine operatory proficiency. Furthermore, such end points are often dependent more on the lesion and other factors related to a specific case than on the operator’s experience. 

New technologies are consistently being developed and deployed into the clinical arena as a means of improving navigational bronchoscopy and increasing the ability of physicians to access peripheral lung nodules. While many of these technologies rely on pre-procedural CT scans as a means of mapping out navigation paths ahead of time, CT-to-body divergence—which is the difference in the patient’s physiologic state prior to and during the procedure—continues to be a significant limiting factor [15,16]. As such, real-time imaging remains an important tool to guide and confirm catheter placement in navigational bronchoscopy. 

Tomosynthesis is a form of medical imaging that uses X-ray technology to produce detailed images. The tomosynthesis process involves taking multiple low-dose X-ray images from different angles, then using computer software to combine these images into a three-dimensional reconstruction within the body. This reconstruction produces a detailed, layered image that allows for a more precise and accurate view of the targeted area. This increased precision allows for the operator to adjust tools as needed during the procedure. The primary advantage during bronchoscopy is the ability to utilize real-time fluoroscopic images to guide bronchoscopic biopsies of lung nodules. The primary advantage during bronchoscopy is the ability to utilize real-time fluoroscopic images to guide bronchoscopic biopsies of lung nodules [17,18,19,20,21,22]. As the bronchoscope is advanced through the airway, updated target locations can be used to better locate the lesion of interest. By incorporating a real-time imaging modality, the effect of CT-to-body divergence can be minimized. This increased accuracy of navigation will become even more important as the role of transbronchial ablation technologies become more readily available.

While imaging modalities, such as CBCT and augmented fluoroscopy, have been shown to help improve diagnostic yield of peripheral lung nodules during navigational bronchoscopy [23], the increased use of radiation should not be overlooked. The risks associated with radiation exposure are well known with excess radiation exposure leading to increased risk of cancer and the development of other radiation-induced illnesses [24,25]. It is important to note that while the risks associated with radiation exposure should not be overlooked, the benefits of imaging modalities can far outweigh these risks in many cases. However, healthcare systems must be vigilant in their efforts to minimize radiation exposure while still utilizing these valuable tools to provide optimal care for their patients. By measuring the learning curve for these new technologies, healthcare systems can focus resources on operators whose time to proficiency may be suboptimal. The ability to efficiently employ radiologic techniques is vital in reducing overall radiation exposure to both the patient and the operator.

**Figure 1:**
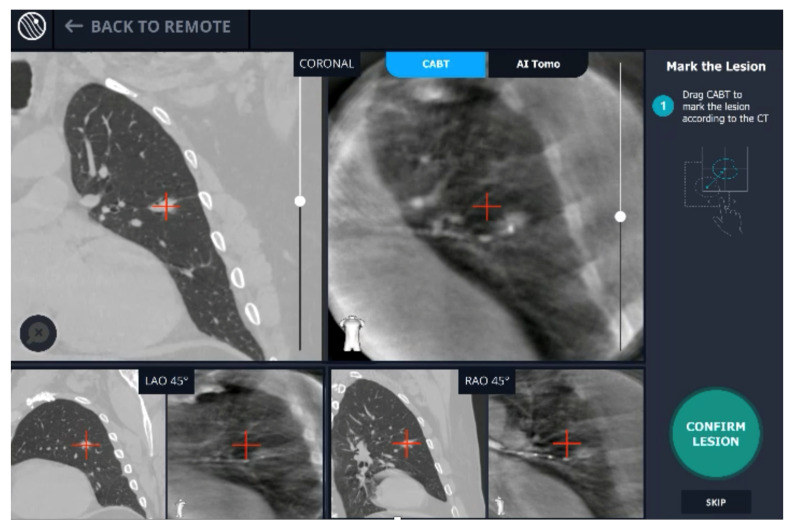
User interface of Body Vision system showing integration of CACT and augmented fluoroscopy.

A new real-time, intraoperative imaging modality was introduced at our academic medical center. Utilizing proprietary, artificial intelligence (AI) algorithms, Body Vision’s C-arm based computed tomography (CABT) technology produces intraoperative three-dimensional imaging from a conventional C-arm that allows for three-dimensional tomographic visualization of the pulmonary lesion during the procedure. It provides augmented fluoroscopy based on this real-time imaging to guide navigation to the actual lesion and provides visual confirmation of “tool-in-lesion” prior to and during biopsy to ensure that tissue sampling occurs from within the lesion (Figure 1). The aim of this study was to evaluate the learning curve of this new modality in terms of the number of procedures required to achieve proficiency. 

## 2. Materials and Methods

### 2.1. Study Design 

This prospective, single-center study evaluated the learning curve associated with early use of the Body Vision platform (Body Vision Medical Ltd., Ramat Hasharon, Israel) by two operators in unselected consecutive navigational bronchoscopy procedures. Body Vision is a novel imaging system that enables intraoperative 3D imaging using a conventional C-arm during bronchoscopy for biopsy of PPLs. The study was conducted in accordance with the International Conference on Harmonization Good Clinical Practice Guidelines and the ethical principles in the Declaration of Helsinki. The study protocol was reviewed and approved by the institutional review board at our academic medical center (IRB Project number 201175). Patients signed an informed consent form before any study procedures were completed. 

### 2.2. Patient Population

Eligible patients were adults >18 years of age with a CT-detected PPL, defined as located at the outer two-thirds of the lung, which were evaluated and deemed appropriate for elective navigational bronchoscopy with biopsy. CT with slice thickness of ≤1.25 mm performed within 30 days prior to the procedure was required and lesions had to be solid or sub-solid measuring 10 mm to 30 mm at the greatest diameter. Patients were excluded if they were participating in any other bronchoscopy studies that could interfere with the quality of data collection, had a bleeding disorder and/or a platelet concentration <50,000 or INR > 2, or were pregnant. 

### 2.3. Study Endpoints

The primary study objective was to measure the number of procedures required to achieve proficiency with the Body Vision system, introduced at our academic medical center in February 2021. Two experienced operators performed the navigational bronchoscopy with biopsy procedures using Body Vision. During these procedures, the onsite manufacturer’s clinical applications representative was available to answer the operator’s procedure-related questions. An independent observer recorded the number of questions asked per procedure. An operator was considered “proficient” when three or fewer procedure-related questions were asked during a procedure and no subsequent procedure exceeded the three-question threshold. Secondary study objectives were: (1) To evaluate tool-at-lesion success rate confirmed by CABT. Tool at lesion was defined as: catheter tip at the edge of the target or within the target as assessed on multiple CABT planes. Tool adjacent to target was defined as tip of catheter within <2 cm from target and oriented towards the lesion on multiple CABT planes. (2) Total procedure time (min) from the time of bronchoscope insertion to the time of bronchoscope removal. (3) Total fluoroscopy time (sec) and radiation dose (mGy/m^2^). (4) Diagnostic yield at end of procedure. Diagnostic biopsies were defined based on final pathology as those that demonstrated a specific malignant process or a specific benign process that clearly explained the etiology. A biopsy that showed inflammation was considered diagnostic only if a subsequent biopsy (surgical or percutaneous) confirmed the benign diagnosis or if the lesion improved or resolved on follow up imaging. (5) Adverse events within 14 days after the procedure. 

### 2.4. Study Procedure 

Pre-procedure CT images were imported into the Body Vision software, where the physician identified the target lesion and selected the planned navigation pathway per manufacturer instructions. All procedures were performed under total intravenous anesthesia without use of inhaled anesthetics. At the beginning of the procedure, an initial CABT (OEC 9900; General Electric, Boston, MA, USA) image centered on the patient’s main carina was performed to correlate the preoperative CT scan with the patient’s positioning during the procedure. A second CABT scan was then obtained to provide an updated, real-time image of lesion location. This CABT-derived target lesion location and pathway were performed as an augmented overlay on live fluoroscopy views throughout the procedure. A flexible bronchoscope (BF-1T180; Olympus Medical Systems Corp., Tokyo, Japan) was navigated to the target lobe and a fluoroscopically visible, steerable catheter (Body Vision Medical Ltd., Ramat Hasharon, Israel) was introduced through the bronchoscope working channel. The catheter was guided to the highlighted target using live, real-time fluoroscopy augmented with an overlay of the pathway to the lesion. When the lesion was reached, a third CABT scan was obtained to confirm catheter location to be within the lesion in multiple orthogonal planes of view or at the edge of the lesion in a location that would allow biopsy without further maneuvers. Once catheter location relative to the lesion was verified, multiple biopsy tools, including a needle, standard cytology brush, biopsy forceps, needle cytology brush, and bronchoalveolar lavage, were implemented according to physician preference. Rapid on-site cytopathologic examination (ROSE) was performed by a cytotechnologist and cytopathologist present in the operating room. 

### 2.5. Statistical Analysis

The sample size and trial duration were primarily determined by feasibility and, although enrollment was originally planned for 50 patients, the study was stopped after 31 cases, as both operators reached defined proficiency. Mean and standard deviation, and median and interquartile range are reported for continuous variables; categorical variables are reported as percentage and counts. Association between variables and the proficiency period was explored. Categorical variables were compared using Chi-square (or Fisher’s exact test), and continuous variables were compared using the Wilcoxon two-sample test. Two-tailed *p* values of less than 0.05 were considered statistically significant. Analyses were performed using SAS 9.4 software (SAS Institute Inc., Cary, NC, USA). 

## 3. Results

Thirty-one patients were enrolled in the study and underwent PPL biopsy using the Body Vision system between February 2021 and August 2021. The patient’s demographics and lesion and procedural characteristics are described in Table 1. The median age was 67 years (IQR 59–78), 45% of patients were female, 68% were ever smokers, and 53% had prior cancer. The mean pre-procedure probability of malignancy based on the Solitary Pulmonary Nodule (SPN) Malignancy Risk Score (Mayo Clinic Model) was 53%. More lesions (71%) were located in the upper lobes than the lower lobes (29%), and nearly all (94%) were in Zone 1. Median lesion size was 16 mm (IQR 12.7–22) with 21 lesions (68%) ≤20 mm, and 10 lesions (32%) >20 mm. Most lesions (84%) were solid, 16% were semi-solid, and none had a ground-glass appearance. 

**Figure 2:**
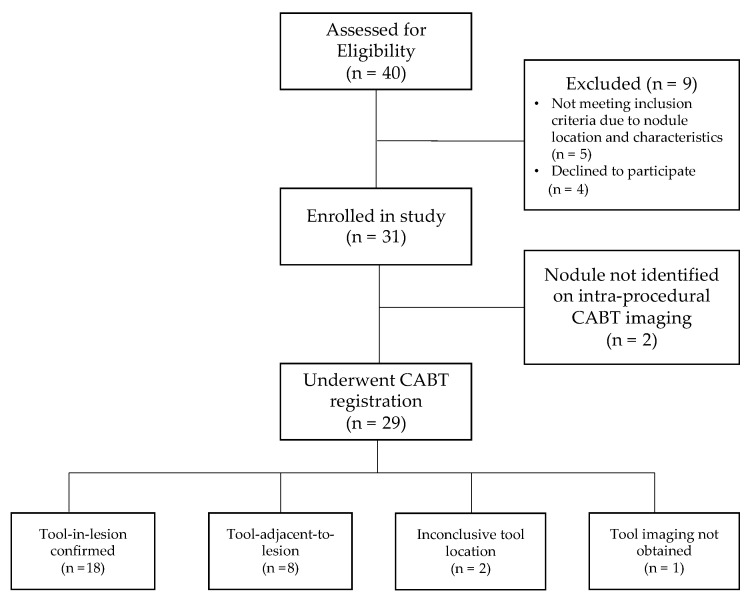
Patient flow diagram.

A total of 31 procedures (18 by Operator 1 and 13 by Operator 2, respectively) were performed in 31 patients with one lesion biopsied in each patient. CABT image registration and confirmation were obtained for 29 patients (93.5%). Of these 29 patients, tool-in-lesion was confirmed by CABT in 18 of 29 patients (62.1%) and tool-adjacent-to-lesion was confirmed in 8 of 29 patients (27.6%); tool location was inconclusive (not localized to lesion) in 2 of 29 patients (6.9%); and in 1 of 29 patients (3.4%) no tool-in-lesion imaging was obtained (Figure 2). On REBUS verification, there was a concentric pattern in 10 patients (32.3%) and an eccentric pattern in 15 patients (48.4%) for a total of 25 of 31 (80.6%) REBUS verifications. In 6 patients (19.4%), we were unable to obtain REBUS confirmation (lack of either a concentric or eccentric pattern). There were no statistically significant differences in patient or lesion characteristics when comparing the two operators for any of the variables outlined in Table 1.

Figure 2 shows the patient flow from recruitment to results of navigation and tool location assessment.

**Figure 3:**
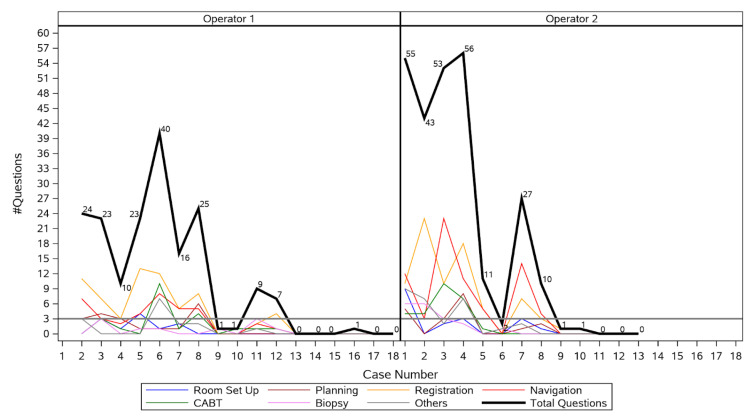
Number of questions asked by each operator during each procedure.

Proficiency was achieved after 12 procedures for Operator 1, and after 8 procedures for Operator 2 (Figure 3), respectively. Hence, the learning curve period encompassed the first 20 procedures, and the post-learning curve period included the next 11 procedures. Operator 1 had a marked reduction in number of questions asked from first to second procedure, passed the three or fewer question threshold after the twelfth procedure, and reached zero questions at the thirteenth procedure. Operator 2 had a marked reduction in number of questions asked from first to fifth procedure, passed the three or fewer question threshold after the eighth procedure, and reached zero questions by the eleventh procedure. The median number of questions asked per procedure decreased significantly from 23 (IQR 9.5–41.5) during the learning curve period to 0 (IQR 0–1) after the learning curve period (*p* < 0.001). Changes in mean procedure time, fluoroscopy time, and radiation dose are shown in Table 2. Median procedure time decreased from 61.5 min (IQR 52.5–74) during the learning curve period to 55 min (IQR 41–69) after the learning curve period (*p* = 0.13). Median fluoroscopy time decreased from 153 s (IQR 113.1–199) during the learning curve period to 132 s (IQR 93.1–149.4) after the learning curve period (*p* = 0.29). Median radiation dose decreased significantly from 19.5 mGy·m^2^ (IQR 1.9–43.5) during the learning curve period to 1.5 mGy·m^2^ [0.7–3.3] after the learning curve period (*p* = 0.05). 

Figure 3 shows that for Operator 1, there was no need for more than 3 questions after the first 12 procedures, whereas for Operator 2, the target of up to 3 questions was obtained after the eighth procedure. During his first procedure, Operator 1 asked an outlier number of questions in total (237), most of which referred to the navigation (67) and biopsy (51). This outlier event was removed from the figure.

The diagnostic yield increased significantly from 65% (13 of 20) during the learning curve period to 100% (11 of 11) after the learning curve period (*p* = 0.03) for an overall diagnostic yield of 77.4% (24 of 31 navigations).

When comparing patient and lesion characteristics between the learning curve and post-learning curve periods, patients in the learning curve period were older (*p* = 0.01) and more lesions were visible with fluoroscopy in the post-learning curve period (*p* = 0.03) (Table 3).

## 4. Discussion

Learning curve studies evaluating navigational and guided bronchoscopy techniques in larger series of procedures (range: 100 to 2042) have utilized a variety of methods to quantify the learning curve with diagnostic yield often being used as a surrogate for procedural success. The operators in these studies run the gamut from residents in training to experienced pulmonary interventionalists, though all were new to using the procedure being studied. Cumulative sum analysis (CUSUM) method, a tool for evaluating the learning curve of a specific procedure, has been used in a study of 215 ENB procedures by experienced interventional pulmonologists [26]. These learning curves based on diagnostic yield demonstrated significant variability between the four operators. Another study of 238 patients undergoing CBCT-guided navigational bronchoscopy with augmented fluoroscopy over a 2.5-year period demonstrated a gradual learning effect with overall improvement in diagnostic yield from 72% to 90% (overall yield 76.4%) and radiation exposure as measured by the dose area product decreasing from 47.5 Gy·cm^2^ to 25.4 Gy·cm^2^ [27]. The improved diagnostic yield was demonstrated in the last 64 patients of the 208 procedures included in the analysis, and the authors did note that some of this change over time may be accounted for by team experience and changes in biopsy protocols. In a retrospective analysis of 100 transbronchial lung cryobiopsy procedures, diagnostic yield increased from 74% in the first 50 procedures to 90% in the second 50 procedures and the learning curve plateaued at the 70th procedure [28]. In a large, retrospective study of 95 robotic bronchoscopies performed over a 14-month period, the learning curve was evaluated by comparing the first 6 months to the last 8 months. The rate of true positives improved from 61% to 82%, and the false negative rate decreased from 12% to 10% (*p* = 0.04) [29]. In a retrospective review of EBUS TBNA procedures over a six-year period, the learning curve appeared to continue beyond 120 procedures [30]. These studies highlight the complexity of using diagnostic yield as a measure of proficiency. 

In our single-center study, 2 experienced bronchoscopists demonstrated proficiency in an average of 10 navigations based on a novel approach to proficiency measurement. They were considered proficient when they asked three or fewer questions of the manufacturer’s representative during a procedure with no subsequent navigations in which they asked more than three questions. This measure of proficiency was supported by secondary workflow endpoints, including a statistically significant 82% reduction in mean radiation dose after the learning curve period, and a clinically meaningful 17% reduction in mean procedure time and a 27% reduction in mean fluoroscopy time. Additionally, there was a statistically significant improvement in clinical outcomes as measured by an increase in diagnostic yield from 65% during the learning curve period to 100% after the learning curve period. 

Procedure time is often linked to proficiency, with the expectation that operators get faster as they become more skilled at performing a given procedure. While this is often the case, procedure time is also impacted by factors, such as procedural complexity, and the difficulty or variable features of the lesion, which may account for the lack of a statistically significant differences in mean procedure time (reduction of 11 min) and, in turn, mean fluoroscopy time (reduction of 51 s); though these decreases should be considered clinically meaningful and may have demonstrated statistical significance in a larger study. The number of questions asked of the manufacturer’s clinical applications representative may be less influenced by these factors, making it a valuable addition to the determination of proficiency. By counting the number of questions during each case, we were also able to measure the operator’s increasing comfort level with using this novel system over time and we could break this down by procedure stages to identify specific steps that may be more challenging.

The statistically significant decrease in radiation dose by 23 mGy·m^2^ is an important improvement for both operators and patients. Given the rising frequency of chest CT and subsequent biopsy procedures for incidental pulmonary lesions, the ability to limit radiation exposure is paramount. A study from a large integrated health system found that between 2006 and 2012, the annual rate of chest CT increased from 15.4 to 20.7 per 1000 person-years, and the annual rate of pulmonary nodule identification increased from 3.9 to 6.6 per 1000 person years [31]. With this increasing number of procedures being performed for solitary pulmonary lesions and the increasing implementation of CT and fluoroscopic navigational adjuncts, lower radiation exposure for operators, assistants, and patients is important. 

Diagnostic yield refers to the proportion of patients who undergo the procedure and receive a definitive diagnosis related to their pulmonary nodule. This yield is related to many factors, including nodule size and location. However, successful use of the device is not always reflected in the diagnostic yield. Although we did demonstrate a significant increase in diagnostic yield after obtaining proficiency, our study was not designed or powered to test the effect of proficiency on diagnostic yield while controlling for other factors that may be related to the outcome. It is clear that there is neither a simple nor standardized methodology, be it procedural or outcomes-based, to assess the learning curves associated with new technologies in the navigational bronchoscopy arena.

Our study was limited by the small number of operators performing relatively few procedures. This limitation may impact its generalizability to the wider population, including more and less experienced operators. Additionally, it was performed at a high-volume academic center, which may have reduced time to proficiency given a potentially higher concentration of procedures over a shorter period of time than other practice locations. Acknowledging these limitations, our study was prospective and introduced a novel measure of proficiency for PPL biopsy procedures that we believe better insulates the measure of proficiency from the impact of case-to-case variability than procedural and outcomes-based measures that have been used historically. The operators were experienced pulmonary interventionalists working in a high-volume, academic pulmonology center where they were able to perform the procedures close together in time, which could have enhanced knowledge retention and, thereby, time to proficiency. A larger study that is statistically powered to detect differences in procedure time is needed to confirm our findings regarding time to proficiency with the Body Vision platform, and to validate the number of procedural questions as a measure of procedural proficiency.

## 5. Conclusions

As new technologies are introduced, the ability to quickly assess proficiency is key to ensuring safe and effective care is provided to patients. Analyzing learning curves offers a key tool that can be employed as a means of identifying an operator’s proficiency with new medical procedures. This study describes a unique and clinically relevant measure to assess proficiency using a new medical device, by plotting the learning curve based on the number of questions asked of a device representative by the operator. By isolating the learning curve from procedural variables, such as lesion size and location, this measure can be more readily and quickly be used to determine proficiency. The difference in clinical outcomes during and after the learning curve period of proficiency shows how critical a robust training program with the support of the manufacturer’s clinical applications representatives can be in accelerating proficiency, shortening the learning curve, and reducing the time to achieving optimal clinical outcomes.

## Figures and Tables

**Table 1:** Patient demographics and lesion characteristics.

Characteristics	Total (N = 31)
Age	68.1 (12.5) [67, 59–78]
Gender—Female	14 (45.2%)
Smoking status	
No	10 (32.3%)
Former	19 (61.3%)
Current	2 (6.5%)
No. of Pack Years	37.9 (27.7) [30, 20–60]
History of cancers	16 (53.3%)
Previous lung interventions/surgery	5 (16.1%)
Lobe	
Left Lower Lobe	3 (9.7%)
Left Upper Lobe	11 (35.5%)
Right Lower Lobe	3 (9.7%)
Right Middle Lobe	3 (9.7%)
Right Upper Lobe	11 (35.5%)
Lesion Type	
Semi-solid	5 (16.1%)
Solid	26 (83.9%)
Lesion Size	18 (6.2) [16, 12.7–22]
Lesion Size > 20	10 (32.3%)
Presence of ‘bronchus sign’	24 (77.4%)
Lesion visible on registration	29 (93.6%)
Lesion visible on confirmation	29 (93.6%)
Lesion visible fluoroscopically	24 (77.4%)
Tool in relation to lesion on confirmation	
Inconclusive	2 (7.1%)
Tool adjacent to lesion	8 (28.6%)
Tool at lesion	18 (64.3%)
REBUS Verification	25 (80.7%)
REBUS View	
Concentric	10 (40%)
Eccentric	15 (60%)

Continuous variables are presented with mean (SD) [median, IQR].

**Table 2:** Procedure time, radiation exposure, and diagnostic yield by proficiency period *.

	Proficiency		*p* Value
	No (n = 20)	Yes (n = 11)	
Total procedure time (min)	67.3 (28.3) [61.5, 52.5–74]	56.2 (21.3) [55, 41–69]	0.13
Fluoroscopy time (sec)	187.9 (162.2) [153, 113.1–199]	137.1 (45.5) [132, 93.1–149.4]	0.29
Fluoroscopy dose (mGy·m^2^)	28.9 (32.9) [19.5, 1.9–43.5]	5.2 (8.4) [1.5, 0.7–3.3]	0.05
Definitive diagnosis	13 (65%)	11 (100%)	0.03

Continuous variables are presented with mean (SD) and [median, IQR]. Categorical variables were compared using Chi-square (or Fisher’s exact test), and continuous variables were compared using Wilcoxon two-sample test. * Operator 1 proficient at >12 cases, Operator 2 proficient at >8 cases.

**Table 3:** Patient and procedure characteristics by period *.

	Proficiency		*p* Value
	No (n = 20)	Yes (n = 11)	
Age	72.3 (11.1)	60.5 (11.7)	0.01
Gender—female	7 (35%)	7 (63.64%)	0.15
Smoking status			0.17
No	4 (20%)	6 (54.55%)	
Former	14 (70%)	5 (45.45%)	
Current	2 (10%)	0 (0%)	
No. of Pack Years	32.3 (22.8)	53.6 (36.6)	0.18
History of cancers	8 (4.11%)	8 (72.73%)	0.14
Previous lung interventions/surgery	5 (25%)	0 (0%)	0.13
Lobe			>0.99
Left lower lobe	2 (10%)	1 (9.09%)	
Left upper lobe	7 (35%)	4 (36.36%)	
Right lower lobe	2 (10%)	1 (9.09%)	
Right middle lobe	2 (10%)	1 (9.09%)	
Right upper lobe	7 (35%)	4 (36.36%)	
Lesion type			0.63
Semi-solid	4 (20%)	1 (9.09%)	
Solid	16 (80%)	10 (90.91%)	
Lesion size	18.1 (5.8) [16.0, 12.9–23]	17.7 (7.1) [18.0, 11–20]	0.69
Presence of ‘bronchus sign’	16 (80%)	8 (72.73%)	0.68
Lesion visible on registration	18 (90%)	11 (100%)	0.53
Lesion visible on confirmation	18 (90%)	11 (100%)	0.53
Lesion visible fluoroscopically	13 (65%)	11 (100%)	0.03
Tool in relation to lesion on confirmation			0.84
Inconclusive	2 (11.11%)	0 (0%)	
Tool adjacent to lesion	5 (27.78%)	3 (30%)	
Tool at lesion	11 (61.11%)	7 (70%)	
REBUS verification	15 (75%)	10 (90.91%)	0.38
REBUS view			0.12
Concentric	4 (26.67%)	6 (60%)	
Eccentric	11 (73.33%)	4 (40%)	

Continuous variables are presented with mean (SD) [median, IQR]. Categorical variables were compared using Chi-square (or Fisher’s exact test), and continuous variables were compared using Wilcoxon two-sample test. Radial endobronchial ultrasound (REBUS). * Operator 1 proficient at >12 cases, Operator 2 proficient at >8 cases.

## Data Availability

Study data is available upon request.
